# Epidemiological Aspects of Human Brucellosis and Leptospirosis Outbreaks in Korea

**DOI:** 10.4021/jocmr630e

**Published:** 2011-07-26

**Authors:** Yangho Jang, Hyobi Kim, Hyung-Ae Bang, Myong-jin Lee, Nong-hun Che, Won-Chang Lee

**Affiliations:** a College of Veterinary Medicine, Konkuk University, Seoul (143-760), Korea; b College of Health Science, Korea University, Seoul (136-703), Korea; c Public health in Department of Nutritional Sciences, Otemae College of Nutrition, Osaka, Japan

## Abstract

In order to compare the epidemiological aspects of human brucellosis (HB) and human leptospirosis (HL) outbreaks in Korea, we have analyzed the current state both of the disease incidence and related risk factors. A total 651 cases of HB occurred between 2001 and 2010 in Korea, and the average incidence rate per 100,000 populations was 0.15. A total 1,153 of HL cases occurred during the same period, and its rate was 0.24. While most of the HB prevalence occurred in the whole year round, prevalence in summer was more prominent for HB (p < 0.01), while outbreaks in autumn was more frequent for HL (p < 0.01). Geographical distribution HB cases were eastern and western regions of the rural (88.6% of total) in the Korean peninsula, showing higher outbreaks than other areas, while HL occurred in easterly regions (64.5%). Significantly more males were infected in both HB (86.8%) and HL (59.5%) than those of females in both HB (13.1%) and HL (41.1%), respectively (p < 0.01). The distribution by age groups were different between HB and HL, while the outbreaks over 62.1% of the cases of HB occurred in 40- to 59-year-old age group, and that of HL was clearly showing a high incidence in the elderly age over 60-year-old (59.6%) (p < 0.01). In both diseases, elderly people especially in famers showed a very high incidence rate (57.9% of HB and 52.2% of HL), which is possible due to increased outdoor activities and a decreased number of young people in those areas. The occupational distribution of HB cases was famers, veterinarians, dairyman and others, and those of HL cases were broad. In conclusion, the difference between HB and HL risk factors reflects the different influence of host/vector, climate, and geographical and environmental characteristics in the epidemiological patterns.

**Keywords:**

Human brucellosis and leptospirosis; Epidemic aspects; Risk factors

## Introduction

In recent years in Korea, human brucellosis (HB) and human leptospirosis (HL) become the major zoonoses with a dramatic increase cases incidence in man. HB is one of the world's major zoonoses that still of veterinarians, public health and economic concern in many parts of the world. Control of brucellosis in livestock is a prerequisite for the prevention of this disease in human beings [1, 2]. Many new, emerging and reemerging diseases of humans are caused by pathogen which originated from animals or products of animal origin. A wide variety of animal species, both domestic and wild, act as reservoirs [3]. In Korea, bovine brucellosis, an infection with *Brucella abortus*, has been investigated in dairy cattle since 1956 [4], and that of HB in 2002 [5]. Leptospirosis is an infection in rodents and other wild domesticated species. Rodents are implication most often in human cases [6]. In case of Korea, after 1984 almost 100 cases of HL were annually reported of the rural in Korean peninsula. Therefore, HB and HL since it was classified and controlled as type 3 notifiable disease in "Communicable Disease Prevention Act of Korea" in 2000 [5].

In the present descriptive study, we investigated the epidemiological aspects of HB and HL outbreaks in Korea, and compared the risk factors between HB and HL during the period of 2001 to 2010.

## Methods

The raw data on investigation and confirmed HB and HL cases in Korea were obtained from the Annual Reports of HB and HL in "Disease Web Statistics System" from 2001 to 2010, Korea Center for Disease Control and Prevention (KCDCP) [7]. To better quantify the impact of HB and HL on health in Korea, we compiled and analyzed information of the cases and rate of prevalence, and related risk factors including frequency distribution of seasonal outbreaks, gender, age, and occupation of the patients. The statistical methods of data analysis included one-way analysis of variance (ANOVA) of the prevalence rate per 100,000 populations between HB and HL. Chi-square test (X^2^-test) was also used to compare frequency rate of the risk factors of the disease between the HB and HL. The analysis was preformed using statistical analysis software by the Microsoft Excel. Result was considered to be statistically significant at p < 0.05 and p < 0.01.

## Results and Discussion

Table 1 shows the prevalence of HB and HL in Korea from 2001 to 2010. A total 651 cases of HB occurred in Korea, and the average prevalence rate per 100,000 populations was 0.15. There are eight species of *Brucella* that are currently recognized based on antigen variation and host specificity. Among those species, *Brucella melitensis* and *B.* suis are the most pathogenic strains for humans. *B. abortus* is less virulent, but it nevertheless causes serious social-economic losses in endemic areas through human exposure to about calves [5]. Increasing cases of brucellosis in cattle have occurred in Korea with the large-scale introduction of under-screened Korean native cattle and the herd test that has been conducted since 2004. As the screening test was reinforced, the number of infected cattle has increased from 1,088 heads in 2003 to 5,380 heads in 2004, and to 26,454 heads in 2006 [8]. Accordingly, the number of HB has also sharply increased from 1 case in 2002 to 215 cases in 2006. A total of 651 cases were reported from 2002 to 2010 [5, 7]. During the same period in HL, the prevalence rate was 0.24 per 100,000 populations with a variation of 0.17 to 0.43. The prevalence rate in HL was much higher than that of HB. This significant difference between HB and HL can be explained on the basis of the difference in their epidemic pattern.

**Table 1:** Comparative Observation of Seasonal and Region Outbreaks Between Human Brucellosis and Leptospirosis in Korea, 2001-2010

Item	Brucellosis		Leptospirosis	
	Cases (%)	95% CI	Cases (%)	95% CI
Season				
Spring	170 (26.1) **	22.2-29.5	32 (2.8)	1.9-3.8
Summer	207 (31.8) **	28.2-35.4	80 (6.9)	5.4-8.4
Autumn	156 (24.0)	20.7-27.3	940 (84.5) **	79.3-83.7
Winter	118 (18.1) **	15.1-21.1	101 (8.8)	7.2-10.4
Total	651		1,153	
Region				
Eastern	256 (39.3)	35.1-43.1	775 (67.2) **	64.5-69.9
Western	321 (49.3) **	45.5-53.1	325 (28.2)	25.6-30.8
Central	67 (10.3) **	8.0-12.6	51 (4.4)	3.2-5.6
Jeju-Island	7 (1.1)	0.3-1.9	2 (0.2)	-
Total	651		1,153	

Average prevalence rate per 100,000 populations from 2001 to 2010. Chi-square analysis indicated a significant difference from the total value. *p < 0.05, **p < 0.01.

The distribution of HB and HL throughout the year revealed that outbreaks in spring to summer (26.1% and 31.8% of total cases) were more frequent in HB than HL (2.8% and 6.9%) (p < 0.01), and HB occurred all year round, although monthly changes in the number of cases occurred. In the case of HL, however, the outbreaks in autumn (84.5% of total cases) was much higher than that of HB (24.0%) (p < 0.01). These data strongly indicate that the incidence is influenced by their peculiar climate, geographical and environmental condition of the epidemiological pattern and aspects [2, 3, 6]. Geographical distribution of HB cases was eastern and western regions of the rural (88.6% of total) in Korean peninsula, showed higher outbreaks than the other area, while HL was occurred in easterly regions (67.2%). As shown Table 2, a significantly more males infected in both HB (86.8%) and HL (59.5%) than those of females in both HB (15.2%) and HL (40.5%), respectively (p < 0.01). These remarkable differences in gender distribution are believed to differences between male and female in terms of works and hosts activities [3, 6]. The percentage distribution of HB and HL cases by age group was as follows: in cases of HB, age groups of under 19, 20-39, 40-59 and over 60 years old the percentage were 0.5%, 13.5%, 62.1% and 23.8%, respectively, while in that of HL cases were 1.2%, 10.3%, 28.9% and 59.6%, respectively. However, the distribution by age groups was different between HB and HL, while the incidences over 62.1% of the cases of HB were occurred in 40- to 59-year-old age group, and that of HL was clearly showing a high incidence in the elderly age that over 60-year-old (59.6%) (p < 0.01).

**Table 2:** Comparative Observation Between Human Brucellosis And Leptospirosis by Gender, Age and Occupation in Korea, 2001-2010

Item	Brucellosis		Leptospirosis	
	Cases (%)	95% CI	Cases (%)	95% CI
Gender				
Male	565 (86.8) **	84.2-89.4	684 (59.5)**	56.7-62.4
Female	86 (15.2)	12.4-17.9	467 (40.5)	37.7-43.3
Total	651		1,153	
Age				
<19	4 (0.5)	-	14 (1.2)	0.9-1.5
20-39	88 (13.5)*	10.9-16.2	119 (10.3)	8.0-12.6
40-59	404 (62.1)**	58.4-65.8	333 (28.9)	26.3-31.5
>60	155 (23.8)	20.5-27.1	687 (59.6)**	57.3-61.9
Total	651		1,153	
Occupation				
Clerk/Servicemen	5 (0.8)	0.1-1.5	44 (3.8)**	2.7-4.9
Salesman	3 (0.5)	-	8 (0.7)	0.2-1.2
Labor	6 (0.9)	0.2-1.6	15 (1.3)	0.7-1.9
Engineers	6 (0.9)	0.2-1.6	15 (1.3)	0.7-1.9
Mistress	8 (1.2)	0.4-2.0	68 (5.9)**	4.5-7.3
Students	2 (0.3)	-	15 (1.3)*	0.7-1.9
Soldiers	0	-	8 (0.7)	0.2-1.2
Farmers	377 (57.9) *	54.1-61.7	602 (52.2)	50.7-53.7
Vet. and others	40 (6.1)	4.3-7.9	0	-
Jobless	8 (1.2)	0.4-2.0	161 (14.0)**	12.0-16.0
Others	196 (30.1)**	26.7-33.6	217 (18.8)	16.6-21.1
Total	651		1,153	

95% CI: Confidence interval of 95%, Chi-square analysis indicated a significant difference from the total value.*p < 0.05, **p < 0.01.

The distribution of HB cases by occupation was as follows: farmers, veterinarians and others, and the others, the percentages were 57.9%, 6.1%, and 30.1%, respectively. However, the distribution by occupational groups was broadly in HL as follows: famers, jobless, mistress, clerk and servicemen, labor, students, engineers, salesman, soldiers, and the others, the percentages were 52.2%, 14.0%, 5.9%, 3.8%, 1.3%, 1.3%, 1.3%, 0.7%, 0.7%, and 18.8%, respectively. In Korea, recently, most young people move to cities for work and elderly people are left to work as farmers on their own land [9]. Thus, the higher incidence in the elderly of farmers may be caused by increased risk of infection due to their engaging in increased outdoor activities in rural areas.

Despite the widespread infelicity of HB and HL, prevention of focal transmission in high-risk areas, and insanitary workplace is necessary. Transmission can be minimized through the health education of residents and travelers in high risk areas, recommending for public health and sanitary education of farmers, protective clothes or masks, limiting access to endemic areas, or taking other appropriate prophylactic measures [3, 6, 9].

In conclusion, this is comparative observation of epidemiological aspects for HB and HL in Korea during the period of 2001 to 2010. The difference in HB and HL risk factors reflects the different influences of hosts/vector, climate, and geographical and environmental characteristics in the epidemiological patterns. It is hoped that this information will be a useful reference in the further studies of HB and HL for the public health service.
